# Mechanisms and impact of genetic recombination in the evolution of *Streptococcus pneumoniae*

**DOI:** 10.1016/j.csbj.2015.03.007

**Published:** 2015-04-08

**Authors:** Chrispin Chaguza, Jennifer E. Cornick, Dean B. Everett

**Affiliations:** Department of Clinical Infection, Microbiology and Immunology, Institute of Infection and Global Health, University of Liverpool, L69 7BE Liverpool, UK; Microbes, Immunity and Vaccines Theme, Malawi-Liverpool-Wellcome Trust Clinical Research Programme, Queen Elizabeth Central Hospital, P/Bag 30096, Blantyre, Malawi

**Keywords:** Genetic recombination, *Streptococcus pneumoniae*, Evolution

## Abstract

*Streptococcus pneumoniae* (the pneumococcus) is a highly recombinogenic bacterium responsible for a high burden of human disease globally. Genetic recombination, a process in which exogenous DNA is acquired and incorporated into its genome, is a key evolutionary mechanism employed by the pneumococcus to rapidly adapt to selective pressures. The rate at which the pneumococcus acquires genetic variation through recombination is much higher than the rate at which the organism acquires variation through spontaneous mutations. This higher rate of variation allows the pneumococcus to circumvent the host innate and adaptive immune responses, escape clinical interventions, including antibiotic therapy and vaccine introduction. The rapid influx of whole genome sequence (WGS) data and the advent of novel analysis methods and powerful computational tools for population genetics and evolution studies has transformed our understanding of how genetic recombination drives pneumococcal adaptation and evolution. Here we discuss how genetic recombination has impacted upon the evolution of the pneumococcus.

## 1 Introduction

Once described as the ‘Captain of the men of death’ by Sir William Osler [1], *Streptococcus pneumoniae* (the pneumococcus) is a gram-positive, aerobic, human-adapted commensal that colonises the human nasopharynx [2]. Pneumococcal carriage rates range from 10 to 80% [3,4]. The wide range of carriage rates is mainly associated with age, with children exhibiting higher carriage than adults [3]. Pneumococcal carriage precedes spread to sterile parts of the body, which results in non-invasive diseases such as otitis media, and invasive pneumococcal diseases (IPD); bacteraemia, pneumonia and meningitis [5,6]. Pneumococcal infections are responsible for approximately one million deaths in children under five annually, of which more than 90% are in resource poor settings, particularly in Sub-Saharan Africa, Latin America and Asia [7]. In addition to geography, additional factors including, age, smoking and co-infection with other diseases such as HIV predispose individuals to pneumococcal infections [8]. At least 93 pneumococcal serotypes are known, based on the structure and antigenicity of the pneumococcal polysaccharide capsule [9–12], a major virulence factor that surrounds the pneumococcal cell wall [13]. Heterogeneity between pneumococcal serotypes has been reported both in terms of the invasive disease potential [14] and global geographical distribution [15]. Pneumococcal Conjugate Vaccines (PCVs) that target the pneumococcal capsule offer protection against the serotypes most commonly associated with invasive disease. PCV7 consists of serotypes 4, 6B, 9V, 14, 18C and 23F whereas the PCV10 consists of the same set of serotypes in PCV7 with the addition of serotypes 1, 5 and 19F. Both PCV7 and PCV10 have been shown to have high efficacy against the vaccine type (VT) serotypes they incorporate and initially led to a dramatic decline in IPD [16,17]. However, in the aftermath of the PCV7 rollout, several studies have reported an increase in the rates of non-vaccine type (NVT) serotypes in carriage [18] and invasive disease [19–22]. The newly introduced PCV13 vaccine, which contains all the serotypes in PCV10 plus 19A, 6B, 7F, promises to further reduce the overall IPD burden by targeting additional serotypes some of which increased in prevalence after the rollout of the previous formulations [17,23].

For *S. pneumoniae* to survive, it essential that it is able to rapidly adapt to clinical interventions and its human hosts' immune responses [24]. The introduction of genomic variation is one of the main mechanisms it can employ to adapt to the host environment, avoid the host immune response and evade clinical interventions. Genetic variation arises in pneumococci and other bacterial genomes as a result of errors by DNA polymerase and the DNA mismatch repair (MMR) system during DNA replication [25]. These alterations in the encoded nucleotide base sequence are known as random mutations. The rate at which random mutations occur varies between bacteria due to the differences in fidelity of DNA polymerases. Spontaneous mutations are either maintained or discarded in the population depending on the fitness cost of the new genotypes [26]. Advantageous genotypes with a lower fitness cost are likely to be favoured by positive Darwinian selection and can rapidly spread and become prevalent within a bacterial population. However, certain genotypes can increase in prevalence purely by linkage to other loci that are undergoing selective sweeps, a process known as genetic hitchhiking [27]. Furthermore within small populations certain genotypes are more likely to become more or less prevalent by chance alone regardless of the fitness costs, this is known as genetic drift. Genomic variation can also arise through lateral transfer of DNA fragments between bacteria followed by integration into the host cell genome, in a process known as genetic recombination or ‘prokaryotic sex’ [28,29]. Numerous studies have demonstrated the contributions of random mutation and recombination to the evolution of bacteria [24,30–33]. However, variations exist in their relative importance depending on the bacterial species or individual strain considered [34]. Together, all the aforementioned processes play a role in the evolution and adaptation of the pneumococcus [35]. In recent years, the rapid generation of WGS data, coupled with the development of efficient analysis tools computational methods have helped to provide better insights into the patterns and dynamics of bacterial recombination.

In bacteria, uptake of exogenous DNA is achieved via three main processes; transformation, conjugation and transduction. Bacterial conjugation occurs when there is direct cell-to-cell contact of the bacteria exchanging DNA via a sex-pilus that protrudes from one cell into the other. During transduction, DNA is transferred from one bacterium to another by viruses that infect bacteria called bacteriophages. Bacterial transformation involves the acquisition of exogenous DNA from the bacterial surroundings followed by the integration of the acquired DNA into the host cell genome [36]. Depending on the diversity between donor and recipient DNA, this can result in the creation of variable or mosaic regions within the chromosomes that exhibit incongruent evolutionary histories with other loci within the chromosomes that originate from multiple strains. In the pneumococcus, uptake of exogenous DNA predominantly occurs through transformation followed by integration into the host cell genome (recombination) [36,37]. DNA uptake is triggered by competence stimulating peptides (CSP) or exported pheromones that work in a quorum-sensing manner [38]. Quorum sensing involves regulation of gene expression in response to in changes in the cell-population density [39]. Induction of competence after DNA damage is considered as an alternative to the SOS system, which is present in other species such as *Bacillus subtilis*, but absent in the pneumococcus [40]. Development of competence and transformation occurs during logarithmic growth of *S. pneumoniae* and requires the expression of key competence (com) genes [41,42]. These include *comC* gene that encodes a CSP and the two-component system (TCS) consisting of a histidine kinase (HK) (*comD*) and a cytoplasmic cognate response regulator (RR) (*comE*) [43]. The TCS activates *comX*, which activates the transcription of a cascade of late competence genes in the pneumococcus [44]. Other dispensable competence genes also help competent pneumococcal cells to outcompete the non-competent cells though triggering autolytic enzymes that kill them [45]. Competent pneumococci can thus acquire DNA released from the killed cells, a process known as fratricide [45]. Conjugative transfers of plasmids have also been documented, however, currently there is no evidence that suggest that phage-mediated transduction occurs in the pneumococcus [46].

Genetic recombination has been extensively studied in *S. pneumoniae* as a model organism. In this review, we discuss some of the recent findings on genetic recombination in the pneumococcus from published literature. We provide an overview of the main mechanisms of DNA uptake and exchange that facilitate pneumococcal recombination with other bacteria using WGS. We also discuss the potential biological and clinical impacts of recombination events in pneumococcal genomes with specific reference to antibiotic resistance, virulence and vaccine evasion.

## 2 Genetic Exchange in Pneumococcal Genomes

*S. pneumoniae* is a naturally competent bacterium, able to actively transport DNA fragments from the environment through the cell wall, into the cell cytoplasm. Transported DNA fragments can then be incorporated into the pneumococcal genome, a process known as genetic recombination [47]. In addition to pneumococcal competence, the simultaneous presence of both a donor and recipient bacterial strain is essential for genetic exchange to occur. Pneumococcal recombination has primarily been reported to occur during nasopharyngeal carriage, chronic polyclonal infection and biofilm formation [48,49]. The nasopharynx is a major reservoir for pneumococcal transmission and thus plays a role in disseminating recombinant bacterium across the human population. There are two main hypotheses to suggest why pneumococcal recombination has predominantly been observed in the nasopharynx. Firstly, the nasopharynx is an environment enriched with other microbial species, in addition to *S. pneumoniae*, which presents the opportunity for genetic exchanges. Secondly, the differential expression of the capsule has been suggested to play a significant role. A thinner capsule is expressed during nasopharyngeal carriage to facilitate attachment to the epithelial surface and this may indirectly allow for easy uptake of DNA as the pneumococcal capsule has been reported to inhibit recombination both *in vivo* and *in vitro* [50]. Additionally, comparative genomic analysis of over 3000 well sampled carriage pneumococci from a refugee camp in Thailand showed that unencapsulated pneumococci had a higher propensity to undergo genetic recombination than encapsulated pneumococci [51].

Lateral (or horizontal) gene transfer of exogenous DNA can result in recombination of either related or unrelated DNA segments. When transformation involves DNA exchange from closely related loci, it is known as homologous (legitimate) recombination. Homologous recombination occurs in the core genome, a subset of genes that are shared and conserved across all members of the species under consideration. Homologous recombination also occurs between mobile genetic elements (MGE) such as insertion sequences (IS), integrons, bacteriophages, plasmids and transposons, considered being part of the accessory genome (non-core genome) [52]. Such recombination exchanges can occur between pneumococci or other closely related oral Streptococci including *Streptococcus mitis* and *Streptococcus oralis* [53]. Homologous recombination can also introduce new genes as exemplified by in molecular biology laboratories where synthetic gene constructs are inserted into the pneumococcal chromosomes [54]. When transformation occurs between unrelated loci, it is known as non-homologous (illegitimate) recombination. The term ‘genetic recombination’ will be used here to refer to all these forms of genetic exchanges.

## 3 Core, Accessory and Pan-genome of *S. pneumoniae*

Pneumococci possess a 2.1 megabases (Mb) pair circular genome that consists of over 2000 predicted protein coding regions and approximately 5% insertion elements [56]. The pneumococcus exhibits a high degree of genomic plasticity as evidenced by the level of genomic variability between isolates, with strains sharing approximately 74% identity at the nucleotide level [55]. On average the core genome of *S. pneumoniae* consists of 1647 predicted coding sequences (including paralogs) [55]. The remaining coding sequences that are not conserved in all members of the species are collectively referred as the ‘accessory’ genome, which usually contain dispensable genes that encode proteins that are not essential to the species. The total gene repertoire available to a species, the combined core and accessory genome, is termed the pan-genome [57]. *S. pneumoniae* consists of an open pan-genome which means that sequencing of new pneumococcal isolates continuously adds novel genes to the current gene pool. Variation in the pneumococcal core genome is predominantly introduced by random mutations and homologous recombination that involves both short and long stretches of DNA, whilst recombination involving unrelated loci is more restricted to the accessory genome. The accessory genome does not contain genes essential to cell survival however it plays an important role in bacterial pathogen evolution [58]. This is largely due to the acquisition of mobile genetic elements that harbour antibiotic resistance determinants and virulence factors [58].

## 4 Capturing Genetic Recombination Signals

Multiple approaches have been employed to identify occurrences of genetic recombination in bacterial genomes, reviewed elsewhere by Posada *et al.* [59]. These range from laboratory methods such as DNA hybridisation, to computational based methods such as Bayesian methods [24,55,60–62]. The Genealogies Unbiased by Recombination in Nucleotide Sequences (Gubbins) software was developed to identify recombination events in closely related pneumococci [63] but it has since been employed to study other bacterial species [64]. Closely related pneumococcal isolates belong to the same sequence clusters (SCs) or lineages. These SCs usually contains a single pneumococcal serogroup or clone inferred by multi-locus sequence typing (MLST). Within such similar isolates, the probability that a single nucleotide polymorphism (SNP) would occur at a specific genomic location, the ‘background SNP density’, is estimated as the total number of SNPs identified in the WGS divided by the overall size of the genome. Whole genome scans are used to determine genomic regions with statistically higher number of SNPs than would be expected by chance. This employs a sliding window approach that involves evaluation of a specified number of nucleotides across the genome. The SNP density within each sliding window is compared to the average background SNP density for the whole genome to determine regions with significantly higher number of SNPs than expected by chance alone [24,63]. Such atypical regions are most likely to have been acquired through genetic recombination rather than spontaneous mutations, which on average introduces 2–4 novel mutations per genome per year. The amount of sequence diversity between donor and recipient strains determines the likelihood that genetic recombination will occur [65]. The true levels of recombination in *S. pneumoniae* and other pathogens are is likely to be underestimated because some events are undetectable [59]. Such events occur between highly similar or identical loci and between distant species [59]. Ancient recombination events that involved distantly related taxa before their divergence into different species are difficult to detect by recombination algorithms because the signals in such loci maybe obscured due to the accumulation of additional point mutations [59].

## 5 Contributions of Recombination and Random Mutations

Beneficial mutations can sometimes arise independently in different bacterial strains. However, competition between strains and Darwinian selection can cause some beneficial mutations to be eliminated or become less prevalent in the bacterial population. This process is known as clonal interference. Genetic recombination provides a mechanism for sustaining such independent beneficial mutations from different strains through combining the different loci that contains the mutations, thus giving rise to recombinant strains that possess both mutations [66]. Although this process has been studied in *Escherichia coli* and *Saccharomyces cerevisiae*, it is presumptive that such mechanisms also occur in *S. pneumoniae* due to its highly recombinant nature [66,67]. The relative contributions of genetic recombination and random mutations to genomic diversifications of bacterial species have been previously reported [33]. A study that used the sequences of the seven MLST housekeeping genes to compare alleles introduced through random mutations and recombination events showed that the recombination to mutation (r/m) ratios varies between species. The r/m ratio measures the total number of SNPs imported from exogenous DNA through recombination (r) to those introduce randomly (m). For the pneumococcus, it was shown that a single nucleotide site is 50-fold more likely to change due to recombination than spontaneous point mutation [33]. Further studies using WGS have shown that genetic recombination in pneumococci is widespread, however, the r/m ratios reported were much lower (~ 7) than observed using MLST sequences [24]. Overall, these results suggest that nearly 90% of all polymorphisms in the pneumococcus have been introduced through recombination exchanges [24]. Further studies have also shown no significant variations in the mutation rates between pneumococcal SCs (lineages); an SC consists of a group of isolates with the same genetic backbone, which might not necessarily represent the same serotype [51]. Overall, an average of 2–4 mutations are introduced within a pneumococcal isolate per year regardless of the SC analysed in the phylogenies [51]. In contrast, every recombination event gives rise to an average of 72 mutations per isolate [24,32] but significant differences exist in levels of genetic recombination (r/m rates) observed between pneumococcal SCs [51].

The pneumococcal polysaccharide capsule is a major pneumococcal virulence factor. However as stated earlier, it is hypothesised to inhibit genetic recombination. New evidence from WGS analyses has shown that the rate of genetic recombination is higher among non-typeable (NT) pneumococci, which do not express a capsule, to encapsulated (typeable) pneumococci [51]. Analysis of pneumococcal strains within a single monophyletic clade consisting of serotype 14 isolates and NTs with the same genetic backbone as serotype 14, showed that the highest rates of genetic exchanges occurred in the NTs [51]. Pneumococcal isolates can undergo capsule switching whereby the serotype of a clone changes due to alteration in the capsule biosynthesis locus or through genetic recombination [68]. Capsule switching between encapsulated and unencapsulated states has been suggested as a transient state via which pneumococci can acquire rapid genetic diversity through recombination without the inhibitive effect of the surface capsule. The genetic diversity acquired through this unencapsulated state would allow pneumococci to evade host immune responses or acquire novel resistance mechanisms that can lead to non-susceptibility to antibiotics upon return to the original or other capsular types [51,69]. Natural capsule switches mediated by recombination at the capsule polysaccharide synthesis (CPS) locus have previously been documented in pneumococci and preceded the introduction of the earliest pneumococcal vaccines [68,70].

**Fig. 1:**
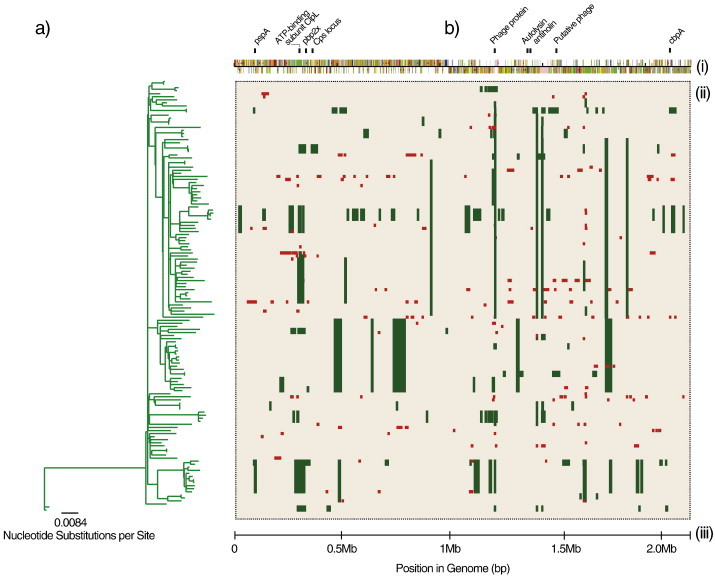
Schematic representation of genetic recombination events in a subset of PMEN1 ST81 *S. pneumoniae* genomes from Croucher et al, (2011), Science. (a) A maximum likelihood phylogenetic tree generated using RAxML version 7.0.3 after removing recombination events in the multiple sequence alignment. (b) Location of genetic recombination events in *S. pneumoniae* genomes. (i) Schematic representation of the sequence features in the *S. pneumoniae* reference sequence. The genes in some regions with high density of recombination events are shown above the features. (ii) Rectangular matrix showing genomic regions where hypothetical genetic recombination events occurred. Recombination events were inferred as regions with atypically high density of polymorphisms. Each horizontal track beginning from the tips of the phylogeny represents a single aligned whole genome pneumococcal sequence. Green blocks shows the presence of a recombination event that was present in at least one isolate (shared) on the same locus while the red blocks represent unique recombination events (different sizes and locations) that were not shared at a particular locus. (iii) Scale showing the position in the genome. (For interpretation of the references to colour in this figure legend, the reader is referred to the web version of this article.)

Recombination events vary in size. Regardless of the pneumococcal serotype considered, genetic recombination events identified in the pneumococcus range from very small fragments to thousands of base pairs (bp) [68,71,72]. Two classes of recombination have been proposed based on the sizes of the recombination events: 1) micro-recombination which involve single and short stretches of DNA occur more frequently and 2) macro-recombination, which are usually rare and consists of multi-fragment replacements of DNA [72]. *In vitro* transformation of the pneumococcus followed by sequencing has shown that average recombination events are ~ 2 kilobases (Kb) [37]. Such *in vitro* experiments coupled with sequence analysis provide invaluable insight into the nature of recombination in the pneumococcus. Analysis of diverse collections of isolates at the population level has shown that on average, the sizes of *in vivo* recombination events are higher than observed *in vitro* using the transformed mutants [24]. In a study of a single lineage of ST81 pneumococci (designated PMEN1 by the Pneumococcal Epidemiology Network) [73], recombination events were found to range from 3 bp to 72Kb with a mean of ~ 6Kb [24]. However, the mean size of recombinant blocks was higher in CC180 (serotype 3) lineage compared to PMEN1 indicating that the distribution of events varies by the lineage considered (Fig. 1) [71]. Overall, the sizes of recombination events in WGS follow a geometric distribution (specifically the exponential distribution) whereby short events are more prevalent than large recombination replacements. The large and multi-fragment recombination events (> 30Kb) have been associated with major phenotype alterations such as capsule switching, which can also result in co-transfer of β-lactam resistance genes located near the CPS locus [68,72].

## 6 Pneumococcal Virulence and Antibiotic Resistance

A landmark experiment on pneumococcal transformation (recombination) by Fred Griffiths is the first and arguably most well-known study to demonstrate the biological importance of this process on pneumococcal virulence [36]. Griffiths demonstrated that genetic recombination through a transforming factor, now known to be DNA [74] from heat inactivated virulent (encapsulated) pneumococci induced virulence in mice that were infected with avirulent (unencapsulated) pneumococcal strains. In addition to demonstrating the biological importance of recombination in virulence and disease, this discovery marked the beginning of the new era of molecular genetics [36].

Genetic recombination plays an important role in the development of antibiotic resistance in the pneumococcus. Antibiotic induced stress is known to induce competence in the pneumococcus; during the competence phase, the pneumococcus acquires exogenous DNA, which may include genes that confer resistance to antibiotics [75]. It has been reported that recombination replacements are responsible for the mosaic structure typically observed in penicillin binding proteins (PBP) genes in *S. pneumoniae* [76]. Mutations in PBP genes confer resistance to β-lactam antibiotics including penicillin, amoxicillin and cefotaxime [77]. β-lactams kill bacteria by inhibiting cell wall biosynthesis and are still by far the most widely used class of antibiotics, as such the increasing rates of resistance in pneumococci are a major global health concern. Recombination with other oral streptococci, particularly the mitis and viridans group played a major role in the initial acquisition of β-lactam resistance in the pneumococcus [79,80]. Recombination involving PBP genes has also been extensively documented to occur between pneumococcal serotypes such as serotypes 9V↔23F, 9N↔14, 7B↔9N, 35C↔17F and 12F↔7F [68,78]. Recombination in *S. pneumoniae* isolates has also been associated with increased levels of resistance to multiple other classes of antibiotics [30]. Recombination mediates the dissemination of transposon and integrative conjugative elements (ICEs) that carry an array of antibiotic resistance determinants, throughout the pneumococcal population [81,82]. Such mobile genetic elements include the Tn*916-*like mobile genetic elements (MGEs), Tn*5251* (a Tn*916-*like element) [83], Tn*5252* [84], Tn*5253* (a composite of Tn*5251* and Tn*5252* transposons) [84] and many others. The Tn*916*, Tn*5251*, Tn*5252*, Tn*5253* and Tn*1545* transposons carry *tetM* gene, which confers resistance to tetracycline [83–86]. In addition to the *tetM* gene, Tn*5251* transposons (the Omega element) also carry the *catQ* gene that confers resistance to chloramphenicol. Another Tn*916* family transposon, Tn*1545*, facilitates resistance to macrolide antibiotics (e.g. erythromycin and azithromycin), mediated by the ribosomal protection gene (*ermB*) and through efflux mechanism (*mefE*) gene that it carries [87]. Several epidemiological studies have reported associations between levels of antibiotic resistance in pneumococcal isolates and the presence of mobile genetic elements, which suggests that genetic recombination involving such MGEs has significant clinical impact [88].

Specific loci accumulate recombination events at a higher rate compared to others. Such regions are known as hotspots of recombination. A recent study of the largest collection of sequenced *S. pneumoniae* genomes to date (*n* = 3085) found an association between genetic recombination hotspots and antibiotic resistance [51]. *S. pneumoniae* isolates that had undergone a recombination in the genes targeted by co-trimoxazole (*folA*) and β-lactam antibiotics (*pbp1a*, *pbp2a* and *pbp2x*) had higher likelihood to exhibit antibiotic resistance to the aforementioned antibiotics [51,89]. In contrast to *tetM* and *catQ* genes, acquired accessory genes, which confer resistance to tetracycline and chloramphenicol respectively, the *folA* and PBP genes are core housekeeping that are not disseminated by MGEs. Recombination encompassing the core genome encoded topoisomerase type II genes has been implicated in fluoroquinolones in both viridans streptococci [90] and salmonella [91], however its contribution to resistance in pneumococci has been reported to be minimal [92,93]. In PMEN1, recombination events and point mutations in the *rpoB* gene were also associated with rifampicin resistance [24]. In addition to *folA* and *PBPs*, other genes reported to be pneumococcal recombination hotspots, are the virulence factors and protein vaccine candidates, *pspA* and *pspC* (*cbpA*) [51]. *pspA* is a cell wall surface protein and a candidate for protein based vaccines and has been shown to increase virulence in mice [94]. *pspC* (*cbpA* or *spsA*) is a choline-binding cell surface protein that plays a role in establishment of colonisation [95]. Thus, the occurrence of genetic recombination in these vaccine candidate and antibiotic resistance genes could result in successful evasion of both the humoral and cell mediated hosts' immune responses and increased levels of resistance to the targeted antibiotics.

## 7 Recombination Drives Vaccine Escape

Since the introduction of PCV7 in developed countries, followed by PCV10 and PCV13, a significant reduction in invasive pneumococcal disease has been reported [96]. PCVs directly work against a set of pneumococcal serotypes whose capsular polysaccharides have been targeted in the vaccine formulation. The introduction of PCVs has led to the emergence of the capsule-switch variants arising due to the vaccine-derived selective pressures [97]. Capsule switching is a natural process that occurs when different pneumococcal serotypes ‘swap’ their capsular polysaccharide locus through alteration of the capsule biosynthesis locus via mutations (single base changes, insertions or deletions) or genetic recombination. When a vaccine-type (VT) strain ‘swaps’ its capsule with a non-vaccine type (NVT) strain, it is able to escape the vaccine since PCVs only confer protection against the VT. An increase in the prevalence of these capsule-switched variants can result in serotype replacement, which is the increase of NVT associated pneumococcal clones, that follows the decrease in VT associated clones [68]. Such replacement of VTs by NVTs has the potential to reduce the impact of vaccination on the overall IPD burden in the long term. Various reports have documented the emergence and circulation of vaccine escape recombinants between serotype VT serotype 19F and NVT serotype 19A following the introduction of PCV7 in the United States [97]. Similar capsule switch variants were also reported in study cohorts from Massachusetts and Thailand [24,32,51]. Apart from capsule-switch, serotype replacement that occurs after introduction of PCVs is mainly caused by ‘serotype unmasking’ [98]. Unmasking is the process where less prevalent or ‘masked’ NVT serotypes rise in prevalence to occupy the ecological niche vacated by the ‘more’ competitive VT serotypes after vaccination [98]. Several reports have shown that serotype replacement is predominantly caused by the unmasking of less prevalent NVT serotypes. Analysis of the genetic background of the replacement serotypes is used to rule out whether such increase of NVT serotypes post-vaccination was caused by capsule switching. For example, comparing antibiotic susceptibility patterns and MLST sequence types (ST) of the replacement serotypes could determine whether or not the same genotypes were present in before the vaccine rollout [99]. Presence of the STs in the replacement serotypes that are associated with different serotypes could suggest that serotype switching has taken place through either point mutations or recombination [99].

## 8 Conclusions

The impact of genetic recombination to the adaptation and evolution of *S. pneumoniae* has been demonstrated by several studies. This has been largely due to parallel advancements in both analysis methods and high throughput sequencing technology. Such advances allow the analyses of whole genome sequences from diverse collections of pneumococcal isolates. Despite these successes, there are still several questions regarding the nature and biological impacts of recombination in pneumococci and other bacterial species. Further studies are required to uncover the exact functional roles of certain recombination events. Most of the genomic studies on pneumococcal recombination have been from a macroevolution standpoint whereby recombination replacements have been analysed at a population level and over large time scales. To better understand the biological roles of these recombination events, future genomic studies should aim to identify the functional roles of certain recombination events in order to provide further insights into pneumococcal pathogenesis, nasopharyngeal carriage dynamics and strain transmission. This knowledge would be invaluable to the development of preventative and control strategies against this important pathogen.
